# Clinicopathological Characteristics of Breast Cancer Patients with Equivocal Immunohistochemistry: A Prevalence-Based Statistical Analysis

**DOI:** 10.30699/ijp.2025.2045071.3378

**Published:** 2025-07-01

**Authors:** Hossein Ayatollahi, Amir Hossein Jafarian, Zahra Pakize Moghadam, Yasamin Ayatollahi, Golnaz Ghayyem Hassankhani, Hassan Mehrad-Majd

**Affiliations:** 1 Cancer Molecular Pathology Research Center, Mashhad University of Medical Sciences, Mashhad, Iran; 2 Pathology Department, Faculty of Medicine, Mashhad University of Medical Sciences, Mashhad, Iran; 3 Cancer Molecular Pathology Research Center, Mashhad University of Medical Sciences, Mashhad, Iran; 4 Mashhad University of Medical Sciences, Mashhad, Iran

**Keywords:** Breast Neoplasms, HER2, In Situ Hybridization, Immunohistochemistry

## Abstract

**Background & Objective:**

Although the concordance between immunohistochemistry (IHC) and fluorescence in situ hybridization (FISH) is generally high for HER2 scores of 3+ and 1+, discrepancies remain in cases scored as IHC 2+. This study aimed to evaluate HER2/neu gene amplification using FISH in breast cancer patients with IHC 2+ and to examine associated clinicopathological characteristics.

**Methods:**

This retrospective study included tissue samples from 369 women diagnosed with invasive ductal carcinoma of the breast and an equivocal HER2 IHC score (2+). These samples were further assessed for HER2 gene amplification using FISH. Demographic and clinicopathological data were collected and analyzed.

**Results:**

The mean age of patients was 51.6 ± 2.6 years. FISH analysis revealed no HER2 amplification in 72.6% of IHC 2+ cases, while 27.4% demonstrated amplification. HER2 amplification was significantly associated with younger age, higher histological grade, lymph node involvement, larger tumor size, and reduced survival rate. No significant association was observed between HER2 amplification and margin involvement.

**Conclusion:**

HER2 amplification is a significant predictor of aggressive tumor behavior and may necessitate targeted therapy. In cases with IHC 2+, both FISH results and relevant clinicopathological features should be considered prior to initiating trastuzumab treatment.

## Introduction

Breast cancer is one of the most prevalent malignancies among women worldwide, accounting for approximately 16% of all cancer-related deaths in women in 2020 (1, 2). If current trends continue, it is projected that by 2040, the number of new breast cancer cases will exceed 3 million annually, and breast cancer-related deaths could rise to 1 million per year (2).

The human epidermal growth factor receptor 2 (HER2) is a transmembrane protein that plays a critical role in the pathogenesis of breast carcinomas (3). Approximately 15% to 25% of breast cancers overexpress HER2 (4). HER2-positive breast cancer is associated with more aggressive clinical behavior, including higher histological grade, early metastasis, and reduced overall survival (4–6).

Trastuzumab, a humanized monoclonal antibody that targets HER2, is widely used in the treatment of HER2-positive breast cancer (4). Clinical trials have shown that administering trastuzumab for one year following adjuvant chemotherapy significantly improves disease-free survival (4). In addition, trastuzumab therapy has been shown to reduce recurrence rates by approximately 50% and to improve survival in advanced-stage disease (7). As a result, current guidelines recommend that all patients diagnosed with invasive breast carcinoma undergo HER2 testing to determine eligibility for trastuzumab therapy (8).

Currently, immunohistochemistry (IHC) and fluorescence in situ hybridization (FISH) are the standard methods for determining HER2 status (8). IHC evaluates HER2 protein expression on the cell membrane, while FISH detects amplification of the HER2 gene (7). Although a high concordance rate exists between IHC and FISH for tumors scored as IHC 3+ (positive) or 1+ (negative), discrepancies are frequently observed in tumors with an IHC score of 2+, which are considered equivocal (9). For such cases, reflex testing with FISH is recommended prior to initiating trastuzumab therapy (10).

Moreover, HER2 gene amplification has been associated with adverse pathological and clinical features in breast cancer (5). Therefore, assessing clinicopathological variables in IHC-equivocal cases may help predict HER2 amplification status. This retrospective study was conducted to determine the prevalence of HER2 gene amplification by FISH in breast cancer patients with equivocal IHC results (IHC 2+) and to evaluate their clinicopathological characteristics.

## Materials and Methods

A total of 369 women diagnosed with invasive ductal carcinoma of the breast and a HER2 IHC score of 2+ between 2014 and 2019 at Ghaem Hospital, Mashhad, Iran, were included in this retrospective study. Patients with missing pathological data were excluded.

The specimens were fixed in 10% neutral-buffered formalin, embedded in paraffin at temperatures below 65°C, and then sectioned into 2 to 4 μm slices using a rotary microtome. IHC testing was performed using the c-erbB-2/HER2-neu (clone SP3) antibody, with heat-induced epitope retrieval carried out using a Bain-Marie water bath. According to the 2024 guidelines of the American Society of Clinical Oncology (ASCO) and the College of American Pathologists (CAP), tissue specimens were scored as negative (IHC 0 and 1+), equivocal (IHC 2+), or positive (IHC 3+).

Score 0 referred to no membrane staining or faint/barely perceptible staining in ≤10% of tumor cells. Score 1+ indicated faint or barely perceptible partial membrane staining in ≤10% of tumor cells. Score 2+ was defined as weak to moderate complete membrane staining in more than 10% of tumor cells, or strong complete membrane staining in ≤10% of tumor cells. Score 3+ denoted strong complete membrane staining in more than 10% of tumor cells.

Samples with an IHC 2+ (equivocal) score were further analyzed by the FISH method. Paraffin-embedded tissue blocks were sectioned into 4 to 6 μm slices, mounted on positively charged slides, and fixed for at least one hour at 60°C. Following deparaffinization, slides were pretreated on a hot plate at 70°C, exposed to xylene, ethanol, and citrate buffer at 98°C to remove aldehyde cross-links. Pepsin solution was then applied, and the slides were incubated at 37°C to digest proteins and expose DNA.

For denaturation and hybridization, the ZytoLight SPEC ERBB2/CEN 17 Dual Color Probe Kit (Z-2020-20, Germany) was used. Samples were denatured at 75°C and hybridized overnight at 37°C. The following day, coverslips were removed, and the specimens were washed in Wash Buffer A at 37°C, dehydrated in ethanol, and counterstained with DAPI/DuraTect solution. Fluorescence microscopy with specific filters for ZyGreen and ZyOrange signals was used to detect amplification of the human ERBB2 gene and chromosome 17 alpha satellites. HER2 amplification by FISH was considered positive if the HER2/CEP17 signal ratio exceeded 2.2.

Demographic data and clinical information including tumor type, grade, size, and lymph node involvement were collected retrospectively. Overall survival (OS) was evaluated via telephone interviews and was defined as the time from breast cancer diagnosis to death from any cause. This study was approved by the Ethics Committee of Mashhad University of Medical Sciences (Ethics code: IR.MUMS.MEDICAL.REC.1403.050), and informed consent was obtained from all participants. Statistical analyses were conducted using IBM SPSS software version 25. Depending on the data distribution, chi-square tests and independent samples t-tests were employed. A p-value of less than 0.05 was considered statistically significant.

## Results

A total of 369 women diagnosed with invasive ductal breast carcinoma and equivocal IHC results were analyzed. Patient and tumor characteristics are presented in Table 1. The mean age of the patients was 51.6 years (standard deviation: 2.6 years; range: 31–65 years). The majority of IHC 2+ cases (72.6%) did not show HER2 amplification on FISH testing, while the remaining 27.4% demonstrated HER2 amplification.

Histological grading revealed that 177 cases (48%) were classified as grade 3, 142 cases (38%) as grade 2, and 50 cases (13.5%) as grade 1. Most tumors (approximately 94%) had negative margins, with only 6% showing positive margin involvement. Lymph node involvement was observed in 325 cases (88%).

Gross pathological examination categorized tumor size as follows: 25 samples (7%) were classified as T1b (>5 mm and ≤10 mm), 130 samples (35%) as T1c (>10 mm and ≤20 mm), 173 samples (47%) as T2 (>20 mm and ≤50 mm), and 41 samples (11%) as T3 (>50 mm), as shown in Table 1.

A statistically significant difference in mean age was observed between patients with and without HER2 amplification. The mean age in the HER2-amplified group was 46.1 ± 6 years, while it was 53.7 ± 6 years in the non-amplified group (p < 0.001). A significant association between tumor grade and HER2 status was also identified (p < 0.001), with more than half of HER2-amplified tumors being classified as histological grade 3.

Lymph node involvement was significantly associated with HER2 amplification status (p = 0.029). Specifically, 94.1% of HER2-amplified cases had lymph node involvement, compared to 86% in the non-amplified group. An inverse correlation between HER2/neu status and overall survival was observed, with HER2-amplified patients demonstrating lower survival rates compared to non-amplified patients (63.4% vs. 73.1%).

Tumor size was also significantly associated with HER2 amplification (p = 0.040). HER2 amplification was most frequently observed in tumors categorized as T2 (>20 mm and ≤50 mm). However, no statistically significant association was found between HER2 amplification and surgical margin involvement (Table 2).

**Table 1:** Clinicopathologic characteristic of patients with invasive breast carcinoma (n: 369)

Variables		No. of patients (%)
Amplification status	Yes	101 (27.4)
	No	268 (72.6)
Histopathological grade	Grade 1	50 (13.5)
	Grade 2	142 (38.4)
	Grade 3	177 (48.1)
Lymph node involvement	Yes	325 (88)
	No	44 (12)
Margin involvement	Yes	21 (5.7)
	No	348 (94.3)
Tumor size	T1b (> 5 mm and ≤ 10 mm)	25 (7)
	T1c (> 10 mm and ≤ 20 mm)	130 (35)
	T2 (> 20 mm and ≤ 50 mm)	173 (47)
	T3 (> 50 mm)	41 (11)
Survival outcome	Alive	260 (70.5)
	Deceased	109 (29.5)
Age (mean, SD)	51.6 (2.6)	

SD: standard deviation

**Table 2:** Correlation of HER-2 status with clinical and histopathologic features in IHC-equivocal patients

Variables		HER2 amplification by FISH method		p-value
		Non-amplified	Amplified	
Age (mean, SD)		53.7 (± 6)	46.1 (±6)	0.000
Histopathological grade (N,%)	Grade 1	45 (16.8)	5 (5)	0.000
	Grade 2	102 (38.1)	40 (39.6)	
	Grade 3	121 (45.1)	56 (55.4)	
Lymph node involvement (N,%)	Yes	230 (85.8)	95 (94.1)	0.029
	No	38 (14.2)	6 (5.9)	
Margin involvement (N,%)	Yes	15 (5.6)	6 (5.9)	0.899
	No	253 (94.4)	95 (94.1)	
Tumor size (N,%)	T1b (> 5 mm and ≤ 10 mm)	19 (7.1)	6 (5.9)	0.040
	T1c (> 10 mm and ≤ 20 mm)	98 (36.6)	32 (31.7)	
	T2 (> 20 mm and ≤ 50 mm)	121 (45.1)	52 (51.5)	
	T3 (> 50 mm)	30 (11.2)	11 (10.9)	
Survival outcome (N,%)	Alive	196 (73.1)	64 (63.4)	0.001
	Deceased	72 (26.9)	37 (36.6)	

## Discussion

The HER2 gene is amplified in 20% to 30% of breast cancer patients. Accurate assessment of HER2 overexpression, either by FISH or IHC, is crucial for predicting patient survival and determining the effectiveness of trastuzumab-targeted therapy. In this retrospective study, we used FISH testing to evaluate HER2/neu gene amplification in patients with IHC 2+ breast tumors. Moreover, we examined the clinicopathological features associated with HER2 status. Our findings demonstrated a statistically significant correlation between HER2 amplification and variables such as age, histological grade, lymph node involvement, tumor size, and survival rate. However, no significant association was observed between HER2 amplification and margin involvement.

In this study, 27.4% of IHC-equivocal cases had HER2/neu gene amplification on FISH testing. These results are consistent with those of Zhang et al., who reported FISH amplification in 29% of patients. Other studies have reported FISH amplification rates ranging from 18% to 23%. A similar study conducted in China found a HER2 amplification rate of approximately 35%, based on Food and Drug Administration (FDA) guidelines. Panjwani et al. reported a significantly higher FISH amplification rate of 67% in IHC 2+ patients. Another study reported that over half of the samples showed HER2 gene amplification, which could be attributed to referral bias. Variation in test results may be explained by differences in tissue processing, fixation duration, the type of anti-HER2 antibody used, or tumor heterogeneity.

According to our data, younger patients are more likely to show HER2 amplification. Previous studies have similarly highlighted that HER2-overexpressing breast cancers are more commonly found in younger women. Anders et al. confirmed that the prevalence of HER2-enriched tumors is higher in younger women. This finding aligns with molecular profiling conducted by Liedtke and colleagues, who examined 4,467 breast tumor cases. Several factors may explain this trend. Firstly, there is a documented interaction between the HER2 and estrogen receptor pathways. Estrogen receptors can regulate HER2 expression in breast cancer cells. The higher prevalence of HER2-positive breast cancers in young women may be attributed to elevated estrogen levels. Moreover, HER2-targeted therapies are less effective in the presence of active estrogen receptor signaling, which can promote cancer cell survival. Secondly, tumor-infiltrating lymphocytes (TILs) significantly influence the tumor microenvironment. Takada et al. suggested that TILs are more abundant in younger individuals, which may promote HER2-driven tumorigenesis. In contrast to earlier findings, however, the correlation between age and HER2 amplification is not consistent across all studies. For example, Al-Zadjali et al. did not find a significant association between age and HER2 status. Since HER2-amplified breast carcinomas in younger populations are more aggressive, it is important to tailor treatment plans to the patient’s age.

A statistically significant association was also observed between FISH amplification status and lymph node involvement. Ninety-four percent of FISH-amplified patients had lymph node involvement. Prior studies have found that HER2-positive breast tumors are more likely to spread to lymph nodes, even at early stages. Moreover, higher HER2 expression levels increase the likelihood of sentinel lymph node (SLN) metastasis. HER2 overexpression is significantly associated with lymph node involvement, and in the presence of lymph node positivity, HER2/neu amplification serves as a stronger prognostic biomarker than hormone receptor status. Studies have suggested that HER2 overexpression is associated with higher vascular endothelial growth factor (VEGF) expression, which promotes lymphangiogenesis and lymph node metastasis. However, in contrast to our findings, some studies did not find an association between HER2 amplification and lymph node positivity.

In this retrospective analysis, we found that HER2/neu amplification was associated with larger tumor size. In a similar study, tumor size was the only clinicopathological characteristic that significantly correlated with HER2/neu amplification. Mostafaa et al. also demonstrated a positive correlation between tumor size and lymph node involvement. Evidence suggests that tumor size is the strongest predictor of axillary lymph node metastasis in patients with breast cancer. HER2 overexpression stimulates tumor cell proliferation and cancer progression by inhibiting apoptosis pathways. The correlation between tumor size and HER2 status highlights the importance of early diagnosis, as smaller tumors typically respond better to treatment.

One of the unique characteristics of HER2-amplified breast cancers is their high histological grade. Our findings revealed that the majority of HER2-amplified tumors had higher grades. Specifically, 39.6% of tumors with amplification were grade 2 and 55.4% were grade 3. These results support the idea that FISH amplification is associated with higher histopathological grades. In general, breast cancers that overexpress HER2 exhibit higher histological grades.

Regarding margin involvement, there was no significant association between margin status and FISH results. A retrospective study on invasive breast cancer patients who underwent lumpectomy also demonstrated no relationship between margin positivity and HER2 status. However, Miller and colleagues observed that a higher percentage of tumors with positive margins showed HER2 expression. They suggested a possible correlation between HER2 overexpression and ductal carcinoma in situ (DCIS) with tumor margin involvement. Nevertheless, our findings indicate that HER2/neu status is not a reliable predictor of margin involvement. In clinical practice, factors such as surgical technique and tumor location have a greater impact on ensuring clear margins.

This study also showed a lower survival rate and a higher mortality rate in patients with amplified HER2 compared to individuals without amplification. In general, HER2 overexpression is closely related to a more aggressive tumor phenotype and worse clinical outcomes. Additionally, clinicopathologic factors such as tumor size, tumor grade, and lymph node involvement can predict survival in breast malignancies. Tumor invasiveness can be attributed to HER2-mediated biological alterations, such as increased lymphangiogenesis and angiogenesis, which raise the likelihood of lymphovascular invasion and metastasis. Available evidence suggests that tumors with both HER2/neu amplification and overexpression are more prone to aggressive behavior than those with either alteration alone. Piccart-Gebhart and colleagues demonstrated that trastuzumab-targeted therapy significantly improved survival in patients with HER2-positive breast cancer. The higher mortality rate in our study may be explained by the limited availability and high cost of targeted therapies in our country.

Several limitations were observed in this study. First, it was conducted using data from a single center, which may not fully represent the broader population. Further studies across multiple medical centers are required to confirm and validate these findings. Second, the retrospective design introduces inherent selection bias.

## Conclusion

This study aimed to evaluate the prevalence of HER2 gene amplification using the FISH method in IHC-equivocal breast cancer cases and to identify associated clinicopathologic features in these patients. Our findings indicate that HER2/neu amplification is significantly associated with lymph node positivity, larger tumor size, higher histological grade, younger patient age, and reduced survival rates. These results underscore the role of HER2 status as a strong predictor of aggressive tumor behavior and the need for targeted therapeutic strategies. Given the potential side effects and substantial costs of targeted treatments, it is essential to consider both FISH test results and the tumor’s clinicopathological characteristics before initiating trastuzumab therapy in patients with IHC 2+ tumors.
